# Evaluating changes in student health, wellbeing and social circumstances before and during COVID-19 pandemic restrictions in Australia

**DOI:** 10.7717/peerj.12078

**Published:** 2021-09-29

**Authors:** Dannielle Post, Agnes Vitry, Katherine L. Baldock

**Affiliations:** 1 Allied Health and Human Performance, Alliance for Research in Exercise, Nutrition and Activity (ARENA), University of South Australia, Adelaide, South Australia, Australia; 2 Clinical and Health Sciences, Australian Centre for Precision Health (ACPreH), University of South Australia, Adelaide, South Australia, Australia; 3 Allied Health and Human Performance, Australian Centre for Precision Health (ACPreH), University of South Australia, Adelaide, South Australia, Australia

**Keywords:** University student wellbeing, Mental health, Social circumstances/determinants, COVID-19, Pharmaceutical and medical science students

## Abstract

The impacts of COVID-19 have been felt on a global scale, with associated physical distancing restrictions and economic downturn having flow-on effects for mental health and wellbeing across the community, and for university students in particular. First-year pharmaceutical and medical science students completing a common introductory population health course at an Australian university are routinely surveyed at the beginning of the semester as part of the course. Survey data inform teaching approaches based on understanding the ‘real life’ commitments and health profiles of students, and deidentified data form part of the teaching material. The 2020 student cohort was invited to complete a second follow-up survey during COVID-19 physical distancing restrictions. A total of *n* = 126 students completed both the initial and follow-up surveys (50.6% response rate), and *n* = 99 (39.8% of the total cohort) consented for their data to be included in research. There was a non-significant decrease in student employment; however, 22% of all students reported loss of work due to COVID-19. There was a statistically significant decrease in the proportion of students undertaking sufficient levels of physical activity, and a statistically significant increase in reported family stressors associated with loss of employment or an inability to gain employment between March and May 2020. Two-thirds of respondents reported increased stress as an impact of the transition to online learning. Implementation of holistic strategies, incorporating attention to additional factors influencing students’ capacity to engage in study, and which may have long-term impacts across the life of the degree program, should be considered.

## Introduction

The impacts of COVID-19 have been felt on a global scale, with the associated social distancing restrictions and economic downturn having a flow-on effect on mental health and wellbeing across all levels of the community. While some positives have been reported anecdotally as a result of social distancing measures, such as the flexibility of working from home, being able to spend more time with household family members, and reduced stress as a result of not having to attend or ferry children to extracurricular activities, there is growing evidence for increases in psychological issues such as anxiety and depression (Fisher et al., 2020).

The negative impact of COVID-19 has been felt in the university sector and for university students in particular (Dodd et al., 2021). This has manifested in many ways, from international students being unable to travel to other countries to attend university, students concerned about graduating on time, first year students adjusting to life at university, and for many students in general, transitioning from on campus, face-to-face modes of delivery to the online learning environment. A study of 1,000 Greek university students identified increased anxiety, depression, and suicidal thoughts associated with the impact of COVID-19 (Kaparounaki et al., 2020). Similar psychological findings were reported in a study of 2,530 Spanish university staff and students, with greater impact for students compared to staff (Odriozola-González et al., 2020). Evidence for psychological distress and an acute stress response was also demonstrated in a study of 1,442 Chinese health students (Li et al., 2020), and a cross-sectional analysis of 787 Australian university students demonstrated low wellbeing and negative experiences related to online learning (Dodd et al., 2021).

It is anticipated that other factors, such as the loss of casual income due to the associated economic downturn, housing concerns, food insecurity, the need for social distancing and its link to social isolation, and the competing demands of family responsibilities, further exacerbate the capacity of domestic and international students to thrive at university (Defeyter et al., 2020; Morris, Mitchell & Ramia, 2020). Additionally, while the shift to online learning may suit some students, it brings with it concerns around equity for students who may not have access to stable internet connections or suitable places to study (Dodd et al., 2021; Hammond et al., 2020).

Since 2015, first year pharmaceutical and medical science students completing a common, on-campus introductory population health course at an Australian university, have been routinely surveyed at the beginning of the semester as part of their enrolment in the course. The survey, largely modelled on national population-based surveys in Australia such as the National Health Survey, collects data about the students’ sociodemographic background, their lifestyle and health behaviours, and chronic disease status. These data are intended to inform teaching approaches that are based on understanding the ‘real life’ commitments and health profiles of students, to better provide teaching environments that enhance students’ learning experience and capacity to learn. Additionally, deidentified data forms part of the teaching content for the course, enabling teaching about concepts such as the social determinants of health and practical skills in working with data. In late March 2020, the university ceased face-to-face education, in line with public health recommendations. As a means of understanding the impact of COVID-19 on students’ capacity to engage with university studies during the pandemic, the 2020 student cohort was invited to complete a follow-up survey in the midst of COVID-19 pandemic physical distancing restrictions. This paper describes the outcomes of that survey.

## Material and Methods

Students enrolled in a first-year introductory population health course across the pharmacy and medical science degrees for the first semester of 2020 (*n* = 249), were invited by email, in the first week of teaching at the start of the course (March 2020), to complete the survey routinely administered at the start of this course each year. Students were subsequently invited by email in May 2020 to complete a second online follow-up survey. The emails included information about the specific purpose of each survey and the voluntary nature of survey completion was explained to the students. Consent for both surveys was implied by the completion and submission of the survey by the student. Students were asked to complete the survey within three weeks, with a reminder email sent after 7-10 days. Students indicated their consent for their data to be used for research purposes *via* a single question in each survey. Approval for this study was provided by the University of South Australia’s Human Research Ethics Committee (protocol number: 0000034112).

### Survey characteristics

Students completed two online surveys, the second being slightly adapted from the original yearly-completed survey, to capture the impact of COVID-19 on lifestyle and health behaviours, employment status, and capacity to undertake study. The surveys elicited general sociodemographic information (*e.g.*, age, gender identify, postcode of residence, height and weight), self-rated health, physical activity behaviour (based on the IPAQ (SF) (Craig et al., 2003), smoking status, fruit and vegetable intake, health service and medication use, mental health diagnoses, family stressors, and income and expenses. Questions in the follow-up survey focused on impacts of COVID-19 such as employment, asking students if their employment status had been impacted by COVID-19, and if so, how they were managing the loss of income; and housing, regarding whether their living arrangements had been impacted. Students were asked to identify the specific factors that had impacted their learning experience during COVID-19 restrictions, and to provide information about the effect of the transition from face-to-face to online learning on their studies. An open-ended question asked students to provide information about the positive and negative impacts of COVID-19 on their lifestyle. The remaining questions required students to provide either a yes/no answer (*e.g.*, Do you currently smoke?), a specific numerical value (*e.g.*, How many times did you do moderate physical activity in the last week?), or to select from a series of answers (*e.g.*, Are you currently in any form of paid employment? 1. No, 2. Yes: Full time, 3. Yes: Part time, 4. Yes: Casual). Surveys were piloted with staff and then students prior to administration. The survey is provided as a Supplementary File.

### Data analysis

Quantitative data were described using median and interquartile range (IQR) for continuous data that were not normally distributed, and proportions for categorical data. Differences in sociodemographic and health-related factors between March and May 2020 were examined for paired data using Wilcoxon Signed Rank Test for continuous data and the McNemar test for categorical data. All analyses were performed using IBM SPSS Statistics, version 25. Statistical significance was set at *α* = 0.05.

## Results

A total of *n* = 126 enrolled students completed both the March and the May 2020 online surveys, representing a 50.6% response rate, and *n* = 99 (39.8% of the total cohort) provided consent for their data to be used for research purposes. Respondents had a median age of 18 years (interquartile range (IQR) =2), and 70.3% were female. Sociodemographic characteristics are reported in Table 1.

Almost half (46.5%) of the student cohort were in some form of paid employment at the commencement of semester; however, 22.2% of all respondents reported experiencing loss of employment due to the COVID19 pandemic. It is possible that some students, in contrast, gained employment between the start of semester and May 2020, since there was no statistically significant difference in the proportion of students reporting to be employed between March (46.5%) and May (39.4%) 2020 amongst student respondents.

There was a non-significant decrease in the proportion of students reporting having a regular source of income, and a non-significant increase in the proportion of students receiving money or income from other sources, such as an allowance from parents. A total of 9% of the respondents reported having changed accommodation due to the COVID-19 pandemic, however there was no significant change in the reported expenditure on housing costs. Respondents reported a statistically significant 25% decrease in the amount they spent in total each week on housing, transport, food, entertainment and mobile phone bills (*p* = 0.002).

There was a significant decrease (*p* = 0.004) in the percentage of students being sufficiently physically active and the percentage of students visiting the dentist (*p* = 0.010), and significant increases for family stressors related to ‘not able to get a job’ (*p* = 0.004) and ‘involuntary job loss’ (*p* < 0.001). There was no significant change in psychological factors, nor were there significant changes for other health-related characteristics, health service use, or family stressors (Tables 2 and 3).

**Table 1:** Sociodemographic characteristics (*n* = 99).

	**Survey 1 (Mar 2020)**	**Survey 2 (May 2020)**	
**Sociodemographic factor**	**% or median (IQR)**	**% or median (IQR)**	**p-value** ^1^
Currently in paid employment: Yes	46.5%	39.4%	0.14
Employment loss due to COVID19: Yes	–	22.2%	
Housing situation			
Live with parents/guardians	68.7%	69.7%	1.00
Live independently (alone or with others)	31.3%	30.3%	
Changed accommodation due to COVID19: Yes	–	9.1%	
Have a regular source of income: Yes	49.5%	43.4%	0.29
Receive money or income from other sources (e.g., allowance from parents): Yes	39.4%	47.5%	0.15
Responsible for paying mobile phone account: Yes	51.5%	55.6%	0.23
Average weekly expenditure: transport^2^	$45 (40)	$5 (40)	<0.001
Average weekly expenditure: food^2^	$50 (72)	$50 (90)	0.16
Average weekly expenditure: housing^2^	$0 (164)	$0 (162)	0.25
Average weekly expenditure: entertainment^2^	$20 (60)	$0 (50)	0.08
Average weekly expenditure: mobile phone^2^	$5 (8)	$5 (12)	0.20
Total average weekly expenditure^2^	$200 (271)	$150 (304)	0.002
Total weekly income^2^	$231 (329)	$231 (326)	0.41

**Notes.**

1 *p*-value based on related-samples Wilcoxon Signed Rank Test or McNemar’s test.

2 A total of *n* = 53 students provided complete data on income and expenditure.

**Table 2:** Health-related characteristics (*n* = 99).

	**Survey 1 (Mar 2020)**	**Survey 2 (May 2020)**	
**Health-related factors**	**% or median (IQR)**	**% or median (IQR)**	***p*-value**
Physical activity			
Sufficiently active	44.4%	30.3%	0.004
Insufficiently active / Sedentary	50.5%	63.6%	
*Not stated* ^1^	*5.1%*	*6.1%*	
Vegetables (usual serves per day)	2 (1)	3 (2)	0.35
Fruit (usual serves per day)	2 (1)	2 (1)	0.76
Self-rated health			
Fair or Poor	12.1%	11.1%	0.74
Good, Very Good or Excellent	87.9%	88.9%	
BMI	22.0 (5.1)	21.7 (5.1)	0.54
Family stressors: Have any of these been a problem for you or anyone close to you, during the last 12 months?			
Serious illness	19.2	20.2	1.00
Serious accident	5.1	5.1	1.00
Death of family member or close friend	23.2	25.3	0.79
Mental illness	21.	28.3	0.23
Serious disability	2.0	1.0	1.00
Divorce or separation	11.1	12.1	1.00
Not able to get a job	13.1	27.3	0.004
Involuntary loss of job	4.0	27.3	<0.001
Alcohol or drug-related problem	10.1	8.1	0.75
Witness to violence	5.1	4.0	1.00
Abuse or violent crime	4.0	3.0	1.00
Trouble with the police	2.0	3.0	1.00
Gambling problem	0.0	0.0	NA
Diagnosed mental health conditions			
Stress	12.1%	12.1%	1.00
Anxiety	20.2%	19.2%	1.00
Depression	14.1%	11.1%	0.51
Other mental health condition	3.0%	3.0%	1.00
Currently experiencing diagnosed mental health condition: Yes	20.7%	22.7%	0.75

**Notes.**

1 Category ‘Not stated’ not included in testing differences in proportions between the March and May 2020 surveys.

**Table 3:** Health service use (*n* = 99).

	**Survey 1 (Mar 2020)**	**Survey 2 (May 2020)**	
**Health service use**	**%**	**%**	***p*-value**
In the last 12 months:			
Consulted GP	70.7	67.7	0.51
Consulted specialist	17.2	18.2	1.00
Consulted dentist	56.6	42.4	0.01
Consulted other health professional	22.2	17.2	0.30
Admitted to hospital as an inpatient	5.1	3.0	0.69
Visited an outpatient clinic	4.0	4.0	1.00
Visited emergency/casualty hospital department	7.1	7.1	1.00
Visited day clinic	15.2	12.1	0.58

The most commonly reported COVID-specific factors related to studying in the online environment were decreased motivation (88.89%), feeling isolated from peers (82.83%), increased difficulties in understanding course content (72.73%), and increased stress (66.67%). The most commonly reported impacts of social distancing measures were lack of interaction with peers (85.86%), deferment of practical classes (65.66%), and lack of communication with teaching staff (57.58%) (Table 4).

The open-ended question was completed by nine students (10%). Perceived positive aspects of COVID-19 included being able to spend more time in the fresh air; more time for exercise; preferences for more courses to be available online; having extra time to study due to reduced travel time; and establishing a routine and becoming used to studying at home, albeit accompanied by anxiousness about returning to on-campus learning. Negative perceptions of the impact of COVID-19 related to issues with access to online teaching technologies, that resulted in the student having to withdraw from courses; the home environment not being conducive to study; not being able to maintain an exercise routine due to limited resources; decreased working hours and loss of income; feelings of isolation from fellow students; and juggling parenting responsibilities with study and work responsibilities.

## Discussion

The purpose of this study was to understand the impact of the COVID-19 pandemic and associated factors on the capacity of first year university students to engage with university studies. We believe that this may be one of only a few studies that compares data collected pre-and-mid pandemic in the same cohort of students, and the only one in Australia, where lockdown duration has not been as prolonged as that in the northern hemisphere. The findings would suggest that while there was a non-significant trend for changes in employment and housing arrangements for the students themselves, family stressors associated with loss of employment or an inability to gain employment were significant. The change in these factors in the two-months between completion of each survey likely reflects the impact of COVID-19 on employment circumstances at the global level.

**Table 4:** Impact of COVID-specific factors: transitioning to the online learning environment and social distancing measures (*n* = 99).

**Factor**	**%**
Impact of transition to online learning environment	
Decreased motivation	88.9
Increased motivation	14.1
Increased stress	66.7
Decreased stress	21.2
Increased difficulties in understanding course content	72.7
Decreased difficulties in understanding course content	9.1
Feeling isolated from teaching staff	61.6
Feeling isolated from peers	82.8
Impact of social-distancing measures	
Lack of adequate workspace at home	38.4
Insufficient internet access	25.3
Lack of access to on-campus services (e.g., library, computer pools)	24.2
Lack of communication with teaching staff (e.g., lecturers / tutors)	57.6
Lack of interaction with peers	85.9
Deferment of classes (e.g., practicals)	65.7
Changes to assessments	49.5
Difficulty accessing necessary course material	23.2

Physical activity, in the form of students meeting physical activity guidelines of 150 min of moderate-to-vigorous physical activity per week (UK CMO Guidelines Writing Group, 2019), was significantly reduced in this cohort. This may be due in part to the onset of cooler weather at the time of the pandemic; however, the closure of gymnasiums as well as changes in active transport are also likely to be contributing factors. These findings are similar to those of a UK study, that demonstrated reduced physical activity and increased sedentary time for students, across a nine-month period (Savage et al., 2021). Another study of university students reported a 30% decrease in physical activity compared to students in previous cohorts (Gallo et al., 2020). The significant change in students consulting the dentist may be due to the six-to-twelve monthly cycle of such visits; however, it is likely that this was due to restrictions outlined by the Australian Government, including deferment of all routine dental examinations and treatment (Australian Health Protection Principal Committee, 2020).

There was no evidence of change in dietary behaviour nor was there identification of food insecurity in this cohort; however, almost 70% of respondents live with their parents which may be a protective factor. Other research suggests that students living independently are more likely to have low levels of food security (Defeyter et al., 2020), and a Canadian study has provided evidence that university students not living with their parents had poorer diets during COVID (Bertrand et al., 2021). These findings also differ from those of Gallo et al. (2020) who found female university students had a higher energy intake than female comparators in the previous year’s cohort and in comparison to their male counterparts in the same cohort.

While there were some positives associated with the pandemic, factors such as decreased motivation, feelings of isolation from peers, and increased difficulties in understanding course content, as well as technological issues, and home environments that are not conducive to study may impact the ability of students to successfully undertake university studies. These factors reflect factors raised in recent studies (Dodd et al., 2021; Hammond et al., 2020). Further, while there were no significant changes in psychological factors in the current study, students did report increased stress. Similar findings demonstrating perceptions of increased stress have been reported in a UK study, although in contrast to our findings, this study also showed significant decreases in mental wellbeing (Savage et al., 2021). This may reflect the ongoing lockdown requirements experienced in the UK, compared to Australia, or may relate to the longer duration between pre-and-post measures in the UK study. Many universities have taken steps to support the study needs of students during COVID-19; however, the implementation of strategies that provide a holistic approach, incorporating the additional factors influencing students’ capacity to engage in study, which may have long-term impacts across the life of the degree program, should be considered.

## Limitations

One limitation of this study is that students were asked to self-report a previous diagnosis of mental illness. Instead, application of a validated tool such as the Kessler-10 (Kessler & Mroczek, 1992) may have provided a more accurate impact of COVID-19 on students’ psychological wellbeing. The small number of students who responded to the open-ended question means that perceptions of the broader negative and positive impacts of COVID-19 are not generalisable to the student population. Further, this study did not distinguish between domestic and international students, which may have impacted the findings. Beyond this, the small sample size predisposes the study to type II error.

## Conclusion

This study has described the impact of transition to the online learning environment, and social distancing measures, as a result of the COVID-19 pandemic. A number of factors have been identified that likely impact the capacity of students to successfully undertake university studies during pandemic-like circumstances. Longitudinal studies should be undertaken to investigate the impact of such factors on the short and longer-term retention of students and students’ achievement at university.

##  Supplemental Information

10.7717/peerj.12078/supp-1 Supplemental Information 1 COVID-19 data timepoint 1 & 2 Survey scores assessing the impact of COVID-19 on health and social factors for first-year university students at two time points.

10.7717/peerj.12078/supp-2 Supplemental Information 2 Blank copy of confidential survey

10.7717/peerj.12078/supp-3 Supplemental Information 3 Code for analysis of COVID-19 dataset

## Additional Information and Declarations

### Data Availability

The following information was supplied regarding data availability: The data are available in the Supplemental Files.
