# Supplementary material for: Evaluating changes in student health, wellbeing and social circumstances before and during COVID-19 pandemic restrictions in Australia
Source: PeerJ. 2021 Sep 29;9:e12078. doi: 10.7717/peerj.12078 (PMC8487240; doi:10.7717/peerj.12078)
Supplement: Supplemental Information 2 [file peerj-09-12078-s002.pdf]

Health & Society Health Survey

Comparing the health of populations requires data and the data are commonly collected via health surveys. In a number of the tutorials we will be working with data to perform calculations, compare groups, and create tables and graphs.

The Health and Society Health Survey includes questions that are used in a number of national Australian surveys of health as well as a number of questions about your previous experience with accessing information and working with data.

Given the many disruptions to life and study created by the current COVID-19 pandemic, we would like to invite you to complete the Health and Society Health Survey a second time. We will share a summary of the results of the second survey, and comparison with the first survey, with all students in the course. All data used from this survey will be **de-identified** (meaning that the data will be made anonymous so no one in your class will be able to associate you with your responses). The de-identified data may be used in future offerings of this course in tutorials and assignments.

We would also like to retain the data collected in the second Health and Society Health Survey for research purposes in order assess the potential impact of COVID-19 on health factors and behaviours by comparing the original survey with the second survey. In any research, all data will be de-identified and you would not be named or identified in any way in resulting conference presentations or publications.

In the survey, there is a specific question seeking your consent to retain and use your de-identified data for future research purposes. Should you choose not to consent to your data being stored, this will not affect your position, now or in the future.

Should you consent to your data being stored and used in future research:

- The data will be stored for the life of the course (Health and Society HLTH 1025).
- Your survey data may be linked to your learning outcomes within the course (assignments and exam) and degree program (grades).
- The data will be stored in electronic form (spreadsheets/database) securely on password protected server managed by the University of South Australia.
- The data will be stored in a re-identifiable (coded) form and the code kept separate to the data.
- All records containing personal information will remain confidential and no information which could lead to identification of any individual will be released, unless required by law.
- The storage of this data for future research purposes has been approved by the University of South Australia's Human Research Ethics Committee. If you have any ethical concerns about the project or questions about your rights as a participant please contact the Executive Officer of this Committee, Tel: +61 8 8302 3118; Email: vicki.allen@unisa.edu.au.

Some of the questions in this survey may raise issues in relation to previous or current experiences. If you feel distressed as a result, we encourage you to seek support through any of the following services:

- UniSA counselling service: https://i.unisa.edu.au/students/studentsupport-services/counselling/
- BeyondBlue: https://www.beyondblue.org.au/get-support
- Your regular General Practitioner.

SELF RATED HEALTH

1. **In general would you say that your health is excellent, very good, good, fair or poor?**
    1. Excellent
    2. Very good
    3. Good
    4. Fair
    5. Poor

PHYSICAL ACTIVITY

1. **In the last week, did you do any exercise which caused a moderate increase in your heart rate or breathing, that is, moderate exercise? (e.g. gentle swimming, social tennis, golf)**
    1. Yes
    2. No
2. **How many times did you do any moderate exercise in the last week?**

[enter number of times]

3. **What was the total amount of time IN MINUTES you spent doing moderate exercise in the last week?**

[enter minutes]

4. **What was the total amount of time IN HOURS you spent doing moderate exercise in the last week?**

[enter hours]

5. **In the last week, did you do any exercise which caused a large increase in your heart rate or breathing, that is, vigorous exercise?** (e.g. jogging, cycling, aerobics, competitive tennis)
    1. Yes
    2. No
6. **How many times did you do any vigorous exercise in the last week?**

[enter number of times]

7. **What was the total amount of time IN MINUTES you spent doing vigorous exercise in the last week?**

[enter minutes]

8. What was the total amount of time IN HOURS you spent doing vigorous exercise in the last week?

[enter hours]

SMOKING STATUS

9. Do you currently smoke?

1. Yes
2. No

10. Have you ever smoked regularly, that is, at least once a day?

1. Yes
2. No

FRUIT AND VEGETABLE INTAKE

The next few questions are about some of the foods that you eat and drink. This question is about your usual consumption of vegetables, including fresh, frozen and tinned vegetables.

A serve of vegetables is defined as half a cup of cooked vegetables, one medium potato or one cup of salad vegetables.

11. How many serves of vegetables do you usually eat each day?

1. 1 serve
2. 2 serves
3. 3 serves
4. 4 serves
5. 5 serves
6. 6 serves or more
7. Less than one serve
8. Don’t eat vegetables

A serve of fruit is defined as one medium piece or two small pieces of fresh fruit, one cup of diced fruit, a quarter of a cup of sultanas, or four dried apricot halves. Fruit juices are not considered to be fruit.

12. How many serves of fruit do you usually eat each day?

1. 1 serve
2. 2 serves
3. 3 serves
4. 4 serves
5. 5 serves
6. 6 serves or more
7. Less than one serve
8. Don’t eat fruit

HEIGHT AND WEIGHT (BODY MASS)

13. What is your height without shoes (in centimetres)?

[enter height]

14. What is your weight undressed in the morning (in kilograms)?

[enter weight]

HEALTH SERVICE & MEDICATION USE

15. Have you taken any of these actions for your own health in the last 12 months?

1. Consulted a GP
2. Consulted a specialist
3. Consulted a dentist
4. Consulted other health professional
5. Admitted to hospital as an inpatient
6. Visited an outpatient clinic
7. Visited emergency/casualty
8. Visited day clinic

16. Are you currently taking any medication that has been prescribed by a doctor?

1. Yes
2. No
3. Don’t know

MENTAL HEALTH

17. Have you ever been told by a doctor that you have any of the following conditions?

1. Anxiety
2. Depression
3. A stress related problem
4. Any other mental health problem
5. None

18. Do you still have any of these conditions?

1. Yes
2. No

MOTOR VEHICLE ACCIDENTS AND INJURY

19. Do you have a current driver's license?

1. Yes – Australian
2. Yes – International
3. No for either Australian or International

20. Have you ever been involved in a motor vehicle accident in which you were the driver?

1. Yes
2. No

21. Have you ever been injured as a result of a motor vehicle accident in which you were the driver?

1. Yes
2. No

FAMILY STRESSORS

22. The next few questions are about things that may have been a problem for you, or family or close friends, during the last 12 months.

Have any of these been a problem for you or anyone close to you, during the last 12 months?

1. Serious illness
2. Serious accident
3. Death of a family member or close friend
4. Mental illness
5. Serious disability
6. Divorce or separation
7. Not able to get a job
8. Involuntary loss of job
9. Alcohol or drug related problems
10. Witness to violence
11. Abuse or violent crime
12. Trouble with the police
13. Gambling problem

COMPUTER SOFTWARE PACKAGE PROFICIENCY

These next questions ask about your basic skills in using a number of software packages.

23. Excel is a software package for managing data. How proficient are you with using Excel?

1. Have never used
2. Have used but do not feel proficient
3. Have basic proficiency
4. Feel very comfortable using Excel

24. PowerPoint is a software package for creating presentations. How proficient are you with using PowerPoint?

1. Have never used
2. Have used but do not feel proficient
3. Have basic proficiency
4. Feel very comfortable using PowerPoint

25. Word is a software package for word processing. How proficient are you with using Word?

1. Have never used
2. Have used but do not feel proficient
3. Have basic proficiency
4. Feel very comfortable using Word

INCOME AND EXPENSES

26. Do you have a regular source of income?

1. Yes
2. No

27. Do you receive money or income from any other sources on a regular basis (e.g., allowance from parents)?

1. Yes
2. No

28. In the last month, what was your total income from all sources?

[enter whole dollar amount]

29. Thinking about your housing situation … Do you live:

1. In a family home with your parent/s or guardian/s
2. In a family home with your children
3. In shared accommodation (e.g., with siblings, friends, housemates)
4. On your own
5. Other

30. Thinking about where you currently live, do you:

1. Pay board to live there
2. Pay rent to live there
3. Live there rent free
4. Make mortgage payments

31. In the last month, how much did you pay towards the cost of your housing (rent/board or mortgage)?

[enter whole dollar amount]

32. Do you own a mobile phone?

1. Yes
2. No

33. Are you responsible for paying a mobile phone account?

1. Yes
2. No

34. In the last month, how much did you pay towards your mobile phone account?
[enter whole dollar amount]

35. In the last week, how much did you spend on transport (including car payments, fuel, bus and taxi fares)?
[enter whole dollar amount]

36. In the last week, how much did you spend on food and non-alcoholic beverages?
[enter whole dollar amount]

37. In the last week, how much did you spend on entertainment (including alcoholic beverages)?

[enter whole dollar amount]

38. Do you have private health insurance? (This includes if you are covered by family health insurance or health insurance included as part of a study visa)
1. Yes
2. No
3. Don't know

COVID-19

The following questions relate to the impact of COVID-19 on your life experiences and university studies in general. They are not specific to your experience with the Health and Society course.

39. Are you currently in any form of paid employment?
1. No
2. Yes – Full time
3. Yes – Part time
4. Yes – Casual

40. Have you lost your employment due to the COVID-19 pandemic?
1. No
2. Yes

41. If yes, how are you managing to overcome the loss of income?
1. JobSeeker payments
2. JobKeeper payments
3. Saved money from entertainment and travel expenses
4. Other (specify)

42. **Have you had to change accommodation due to the COVID-19 pandemic?**
    1. No
    2. Yes

43. **If yes, please specify the reason:**
    1. Preferred to live elsewhere
    2. Preferred to live closer to friends/relatives
    3. Could not afford the rent
    4. Other (specify)

44. **What factors have had an impact on your learning experience due to COVID-19 social distancing measures at UniSA?** (choose all options that apply)
    1. Lack of adequate workspace at home
    2. Insufficient internet access
    3. Lack of access to on-campus services (e.g., library, computer pools)
    4. Lack of communication with teaching staff (e.g., lecturers / tutors)
    5. Lack of interaction with peers
    6. Deferment of classes (e.g., practicals)
    7. Changes to assessments
    8. Difficulty accessing necessary course material

45. **Has the move to online teaching due to COVID-19 affected any of the following factors related to your studies?** (choose all options that apply)
    1. Increased motivation
    2. Decreased motivation
    3. Increased stress
    4. Decreased stress
    5. Increased difficulties in understanding course content
    6. Decreased difficulties in understanding course content
    7. Feeling isolated from teaching staff
    8. Feeling isolated from peers

46. **Is there anything else you'd like to share with us about your experiences during this COVID-19 pandemic? This may relate to either positive or negative effects on your lifestyle?**

    [open response]

SOCIO-DEMOGRAPHICS

47. What is your gender?

1. Female
2. Male

48. What is your age (in full years)?

[enter age]

49. What is the postcode of your current place of residence?

[enter postcode]

FUTURE DATA USE

50. I give my consent for my data to be retained and used in future research

1. Yes
2. No

If you answer yes, your de-identified data will remain in the data set (i.e., your name and student ID will no longer be associated with your health survey data).

If you answer no, your data will be removed from the data set.

If any of the questions in this survey have caused you distress, we encourage you to seek support through any of the following services:

- UniSA counselling service: https://i.unisa.edu.au/students/studentsupport-services/counselling/
- BeyondBlue: https://www.beyondblue.org.au/get-support
- Your regular General Practitioner.
